# Self-navigated free-breathing isotropic 3D whole heart MRI for the characterization of complex cardiac anatomy in patients with congenital heart malformations

**DOI:** 10.1186/1532-429X-15-S1-P12

**Published:** 2013-01-30

**Authors:** Pierre Monney, Davide Piccini, Gabriella Vincenti, Arne Littmann, Michael O Zenge, Simon C Koestner, Matthias Stuber, Juerg Schwitter

**Affiliations:** 1 Division of Cardiology and MR Center, University Hospital (CHUV), Lausanne, Switzerland; 2 Centre d'Imagerie Biomédicale (CIBM) & Center for Cardiovascular Magnetic Resonance Research (CVMR), University of Lausanne, Lausanne, Switzerland; 3 Healthcare Sector, Siemens Schweiz AG, Lausanne, Switzerland; 4 Healthcare Sector MR Application & Workflow Development, Siemens AG, Erlangen, Germany

## Background

Cardiac and vascular anatomy of patients with congenital heart disease (CHD) is often complex and accurate evaluation mandates MR image acquisition in several non-standard planes. This requires significant patient involvement while multiple additional breath-holds are required. For these reasons, the utility of self navigated isotropic 3D-free-breathing whole-heart MRI (SN-3D) was assessed for the visualization of heart, coronary arteries (CA) and great vessels in CHD patients.

## Methods

Twelve patients (>10 years old) with CHD were studied. Data acquisition was performed during free breathing on a 1.5T-MRI scanner (MAGNETOM Aera, Siemens AG) with a previously described 3D-radial trajectory [1] implemented for respiratory self-navigation [1]. All measurements were ECG-triggered, with a T2-preparation pulse and fat-saturated mid-diastolic bSSFP readout. The SN-3D started roughly 4 minutes after injection of 0.2 mmol/kg of gadobutrol. Imaging parameters were: TR/TE 3.1/1.56 ms, FOV (220 mm)^3^, matrix 1923, acquired voxel size (1.15 mm)^3^. The scan was performed over 377 or 610 heartbeats, depending on the patient's heart rate. The isotropic image data were reformatted offline.

## Results

**Figure 1:**
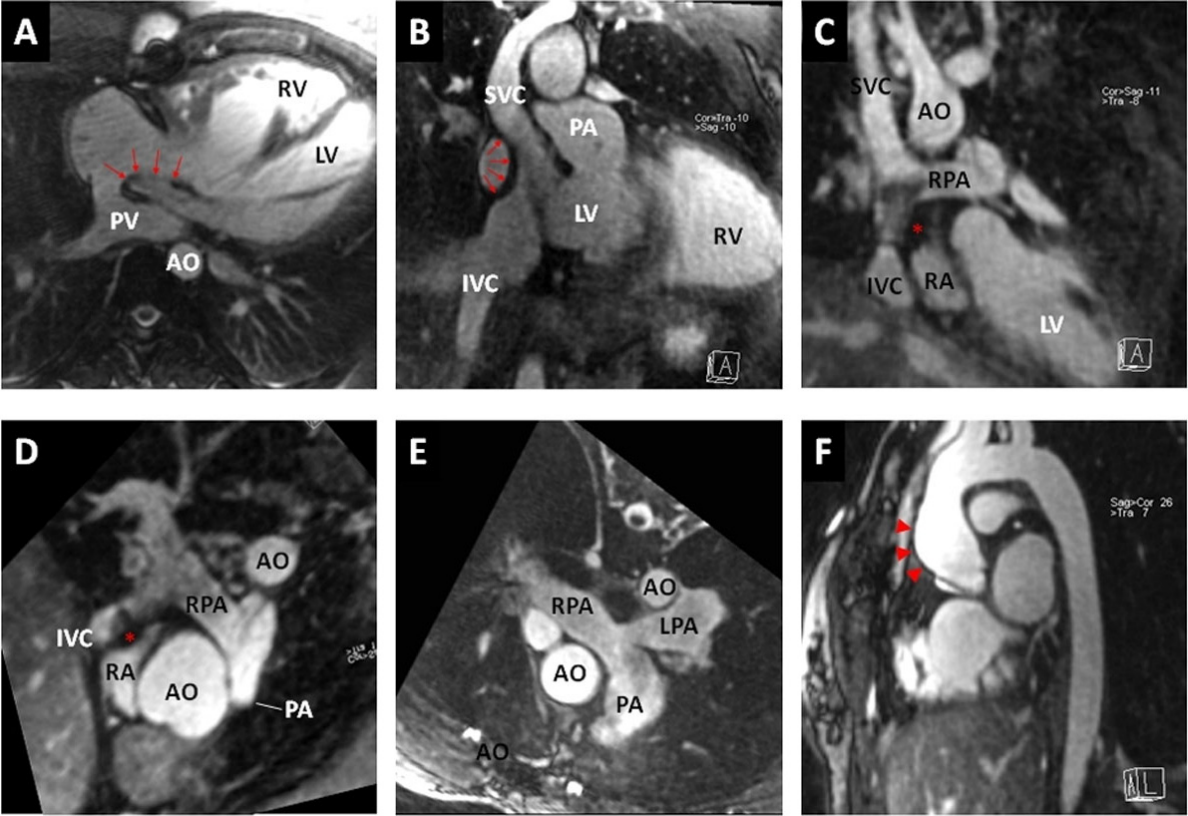
A) Pulmonary pathway connecting the pulmonary veins to the right ventricle after Mustard operation for D-transposition of the great arteries. Red arrows indicate inter-atrial baffle. B) Systemic pathway connecting the caval veins to the left ventricle in the same patient. C) Total cavo-pulmonary connection in a case of pulmonary atresia with intact septum (Fontan circulation). Note a metallic artifact in the inferior cavo-pulmonary conduit (extracardiac conduit) corresponding to a previous percutaneous closure of a fenestration (*). D) Normal anatomy of the branch pulmonary arteries despite hypoplastic pulmonary trunk in the same patient. E) Pulmonary arteries in a corrected tetralogy of Fallot, with mild kinking of the proximal left pulmonary artery. F) Dilatation of the ascending aorta (arrowheads) and "crenel" morphology of the aortic arch in a young girl with Turner syndrome and bicuspid aortic valve.

**Figure 2:**
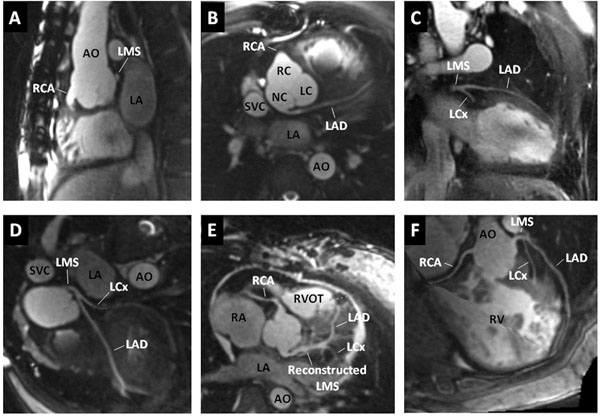
A-C) Abnormal left coronary artery (LCA) arising from the non-coronary sinus and running between the aorta and the left atrium. D) Curved reconstruction of abnormal LCA from the non-coronary sinus. E) Curved reconstruction of a surgically reconstructed left main stem in a case of an abnormal left coronary artery arising from the pulmonary artery (ALCAPA) syndrome. F) Normal course of the coronary arteries in a case of TGA. Aorta is connected to the morphological right ventricle. LMS: left main stem, LAD: left anterior descending artery, LCx: left circumflex artery, RCA: right coronary artery, NC: non-coronary cusp, RC: right coronary cusp, LC: left coronary cusp, LA: left atrium.

The mean duration of the acquisition was 6.3±1.2 min. 3D-isotropic datasets allowed the assessment of the arrangement of cardiac chambers and great vessels, the anatomy of pulmonary (Figure 1A) and systemic venous (Figure 1B) returns after atrial switch for D-transposition of the great arteries, the morphology of cavo-pulmonary connections after Fontan operation (Figure 1C), the morphology and size of the pulmonary arteries in Fontan patients (Figure 1D) or tetralogy of Fallot (Figure 1E), and the morphology of the aorta (Figure 1F). In the high-resolution reformatted images, it was possible to measure the cross-sectional diameters of the great vessels; moreover, the proximal 3 cm of the CA could be well visualized and their 3D course followed in most of the patients (12/12 for LAD, 9/12 for LCx, 11/12 for RCA), making SN-3D useful to detect anomalous courses or connections of the CA (Figure 2A-F).

## Conclusions

The SN-3D methodology enables time-efficient whole-heart coverage during free breathing. The high isotropic resolution supports multi-planar offline reformatting in any plane orientation and allows quickly planning targeted additional flow or cine images, which is particularly useful in CHD patients with complex anatomy. The only determinant for scanning time is the heart rate, while the need for breath-holding is entirely removed. Self-navigation and the absence of fold-over artifacts with the 3D-radial acquisition enhance the ease of use of this sequence and favor a fast acquisition planning. Even small anatomical structures could be precisely identified illustrating the potential of this methodology to assess the anatomy of the proximal course of CA.

## Funding

No funding
